# A COMPARISON OF TRADITIONAL REMOTE MONITORING (RPM) DEVICES VERSUS A NOVEL, TOILET SEAT–BASED MONITORING DEVICE

**DOI:** 10.1093/geroni/igac059.2192

**Published:** 2022-12-20

**Authors:** Carla VandeWeerd, Erica Sappington, Lydia Poon, Chidi Okeahialam-Iheanyi, Mitchell Roberts

**Affiliations:** UFHealth Precision Health Research Center-The Villages, The Villages, Florida, United States; UFHealth Precision Health Research Center-The Villages, The Villages, Florida, United States; UFHealth Precision Health Research Center-The Villages, The Villages, Florida, United States; UFHealth Precision Health Research Center-The Villages, The Villages, Florida, United States; UFHealth Precision Health Research Center-The Villages, The Villages, Florida, United States

## Abstract

As the aging population increases, older adults are disproportionately susceptible to cardiovascular disease(CVD). Routinely monitoring vital signs can alleviate CVD’s burden. Current telemonitoring technologies targeting vital signs can be complex, generating inconsistent outputs and suboptimal adherence. In response, Casana developed the Heart Seat(THS), a passive toilet seat-based remote patient monitoring (RPM) device. This study aimed to compare older adults’ (1) adherence between traditional telemonitoring(TT) devices and THS and (2) satisfaction across devices. A randomized control study (UFIRB202100869) was conducted in an active lifestyle retirement community-The Villages, Florida. Using a crossover design, participants (N=49) with CVD were followed for 12 weeks (8/12/2021-12/21/2021), beginning in TT or THS Arm and crossing over arms at 6 weeks. Adherence was calculated daily as ≥1 use for ≥90 seconds for THS, and ≥1 use of the blood pressure cuff and scale for TT. Descriptive statistics and t-tests were used to evaluate adherence rates and participant’s experience and perception of RPM devices. Participants mean age was 73.4 years (SD=7.1,51% female). The majority (94%) of participants had previous experience using telemonitoring devices (e.g. scale, blood-pressure cuff, smartwatch) and 87.7% strongly agreed to feeling comfortable with at-home health monitoring. Overall, participants demonstrated greater adherence to THS than TT (t(48)=4.86, p<.001). These findings suggest older adults are becoming increasingly experienced and comfortable with at-home monitoring of health parameters. Overall, adherence was greatest in THS, suggesting that passive, easy-to-use telemonitoring devices may increase adherence, promote real-time health monitoring, and support adverse event prevention in older adults.

